# A Porous Geopolymer Containing Ti-Bearing Blast Furnace Slag: Synthesis, Characterization, and Adsorption-Photodegradation Studies towards Methylene Blue Removal under Visible Light Condition

**DOI:** 10.3390/molecules28093673

**Published:** 2023-04-24

**Authors:** Yijian Cheng, Kun Wang, Peng Li, Hongwei Guo, Bingji Yan, Dong Chen, Wei Zhao

**Affiliations:** 1 Shagang School of Iron and Steel, Soochow University, Suzhou 215021, China; chengyijian21@163.com (Y.C.);; 2 State Key Laboratory of Heavy Oil, China University of Petroleum (East China), Qingdao 266580, China; 3 State Key Laboratory of Advanced Metallurgy, University of Science and Technology Beijing, Beijing 100083, China; 4 Key Laboratory of Metallurgical Emission Reduction & Resources Recycling (Anhui University of Technology), Ministry of Education, Maanshan 243002, China

**Keywords:** Ti-bearing blast furnace slag, porous geopolymer, adsorption, photocatalysis, methylene blue

## Abstract

A porous geopolymer with adsorption and photocatalytic degradation functions was successfully developed by utilizing Ti-bearing blast furnace slag (TBBFS) as the raw material. The prepared porous geopolymers were characterized by X-ray diffraction, scanning electron microscope, energy dispersive spectrometer, and Fourier transform infrared spectrum. Selective crystallization, water quenching, and natural cooling methods were employed to investigate the influences of these modifications on the applicability of TBBFS as a precursor for geopolymer synthesis. Water-quenched slag with amorphous content was prone to alkali dissolution, and the resulting geopolymer exhibited the highest adsorption capacity (97.18 mg/g) for methylene blue (MB) removal. Selective crystallization at 1400 °C generated a hybrid microstructure consisting of a non-cementitious CaTiO_3_ crystallization phase and a cementitious amorphous fraction. The retention of CaTiO_3_ in the final geopolymer enables a bifunctionality in adsorption–photodegradation. Particularly, the adsorption and photodegradation processes under various conditions were investigated. The superior removal efficiency for MB could be attributed to the synergistic effects between the geopolymer matrix and CaTiO_3_, leading to an enhancement in the formation of hydroxyl radicals. The conversion of TBBFS into porous geopolymer offers an efficient and straightforward solution for slag utilization and dye removal.

## 1. Introduction

China holds more than half of the world’s total titanium reserves, with approximately 90% located in the Panxi region in the form of vanadium–titanium magnetite [1]. Over 3-million tons of Ti-bearing blast furnace slag (TBBFS) are generated annually when vanadium–titanium magnetite is melted for iron production [2,3]. The inappropriate disposal of TBBFS cannot only be hazardous to the environment but also lead to a potential waste of titanium resources [4]. Recently, the conversion of TBBFS into photocatalytic materials (TiO_2_, CaTiO_3_) has garnered significant attention and can be achieved through alkali fusion and acid leaching [5,6]. Lü et al. [7] converted TBBFS to a visible-light-responsive photocatalyst CaTiO_3_ by thermal treatment with NaNO_3_, followed by HCl leaching. Similarly, Li et al. [8] fabricated nanostructured TiO_2_ photocatalysts with diverse crystal morphologies by subjecting TBBFS to molten NaOH treatment, water leaching, and hydrolysis procedures. Despite numerous successful attempts, the preparation of the photocatalyst mentioned above requires the extraction of Ti from TBBFS via intricate processing stages, which inherently possess drawbacks of high operational and handling costs. Simultaneously, other components inevitably result in highly hazardous sludge and are not fully utilized. Furthermore, due to environmental concerns, the processing sludge presents additional treatment and disposal challenges. Hence, it is crucial to improve the conversion process by developing an eco-friendly and effective methodology for the value-added utilization of TBBFS.

Geopolymers are inorganic polymeric materials synthesized by aluminosilicate sources such as granulated blast furnace slag (GBFS), metakaolin (MK), and fly ash (FA) [9]. In recent years, geopolymers have attracted considerable attention as a new type of sustainable material due to their potential applications in cement, concrete, and environmental remediation [10,11,12]. Porous geopolymers have been proven to be capable of adsorbing toxic and harmful organic substances [9,13]. Furthermore, the introduction of a photocatalyst into geopolymer imparts photocatalytic activity that enables the pollutants to be photodegraded. Maiti et al. [14] successfully prepared a photocatalytic geopolymer by mixing TiO_2_ nanoparticles with fly ash, alkali activator, and normal sands. In addition, to improve the mechanical properties of modified geopolymer, the incorporation of nano TiO_2_ exhibited a considerable removal efficiency of methylene blue (MB) dye (96.4% degradation in 90 min). Li et al. [15] prepared geopolymer spheres by suspension solidification and facilitated the growth of CdS on the surface of geopolymer spheres by van der Waals forces and ion–exchange interactions. The degradation efficiency for methyl orange of the material reaches approximately 92.57%. Falah et al. [16] prepared novel photoactive composites for the destruction of organic pollutants by blending the uncured geopolymer mixture with Cu_2_O/TiO_2_ nanospheres. After 12 h of adsorption in dark and 4 h of photodegradation under UV irradiation, the removal efficiency of MB reached 99%. These studies demonstrated that geopolymers could be employed not only as adsorbents but also combined with semiconductor materials to create a new composite material that can exhibit both adsorption and photocatalytic degradation functions.

TBBFS typically comprises oxides of CaO, SiO_2_, MgO, etc., while the content of TiO_2_ is 12–28 wt.%. TBBFS has the advantage of high Ti content and utilizes Ti components. Previous studies have proven that implementing slag modification combined with selective crystallization allows for the enrichment of Ti in the form of a perovskite phase, while the remaining components exist in an amorphous state [4,17]. Based on this, we proposed a facile and straightforward methodology for manufacturing photocatalytic porous geopolymers from TBBFS. By applying selective crystallization to TBBFS at a high temperature (1400 °C), the solely precipitated CaTiO_3_ can be acted as a promising photocatalytic material [18], while the amorphous glassy components can be employed as a precursor for geopolymer formation. During geopolymerzation, CaTiO_3_ will remain intact due to its high chemical stability against alkali dissolution, thereby providing photocatalytic activity in the resulting geopolymer. Currently, geopolymers are often utilized in organic wastewater treatment in the form of monoliths, spheres, and powders [15,19,20]. However, geopolymer powders exhibit characteristics such as small particles, uniform distribution in wastewater, and rapid action time. Compared to conventional methods, the proposed approach has the advantages of (i) Reduced environmental risk, as no Ti extraction methods are employed; (ii) Full utilization of all components in TBBFS; and (iii) More effective adhesion between CaTiO_3_ and geopolymer matrix compared to simply mixing.

The properties of geopolymer can vary significantly depending on the synthesis parameters, raw materials, and mixed proportions. In the context of slag reactivity, it is reasonable to expect the differences as the aluminosilicate species participate in either glassy or crystalline structure. Hence, in addition to selective crystallization, water quenching and natural cooling were also employed to investigate the influences of these cooling paths on the applicability of TBBFS as a precursor for geopolymer synthesis. Most importantly, these thermal treatments for TBBFS modification are easily scalable to implement at an industrial scale, which is crucial for the practical realization of this promising process. In this study, the effects of processing parameters on the phase transformations, microstructure, and textural properties of the geopolymer were systematically studied. The obtained geopolymer exhibited a high specific surface area, abundant porous structure, and high photocatalytic activity, which were applied for the removal of MB, providing further environmental benefits.

## 2. Results and Discussion

### 2.1. Mineralogy and Reactivity of Modified TBBFS

**Figure 1:**
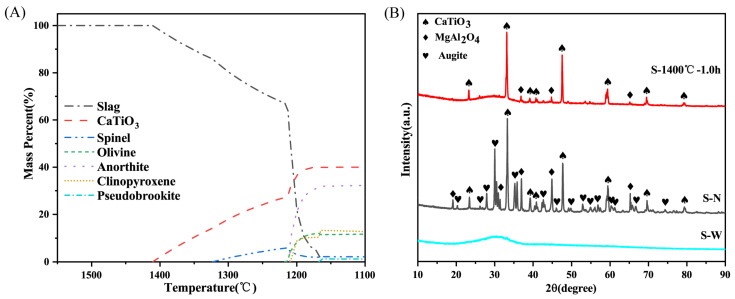
(**A**) The thermodynamics calculations based on equilibrium cooling from 1550 °C to 1100 °C for TBBFS; (**B**) XRD patterns of slags cooled by different ways: S–1400 °C–1.0 h, S–N, and S–W.

Figure 1A illustrates the simulated equilibrium solidification of TBBFS with temperature using Fact–Sage software. The perovskite phase began to precipitate solely at 1410 °C, and its content gradually increased as the temperature decreased. This indicates that the selective crystallization of perovskite can be achieved using the as-received TBBFS without further compositional adjustments. According to the thermodynamic prediction, the optimal temperature for selective crystallization would be close to 1410 °C. The XRD analysis in Figure 1B confirmed that the thermal modification at 1400 °C for 1 h resulted in the precipitation of perovskite from vitreous slag. However, the minor phase crystalline peaks assigned to aluminum spinel MgAl_2_O_4_ (JCPDS 82-2424) also appeared, probably due to its high melting point. In the natural-cooled slag, minerals of perovskite, augite (Ca(Mg,Fe,Al,Ti)(Si,Al)_2_O_6_), and spinel were precipitated. The deviation from predicted mineralogy based on thermodynamic calculations is due to limited mass transfer during the actual cooling process. Conversely, the water-quenched slag underwent a rapid cooling rate, and its mineralogy was predominantly glassy, as no diffraction peaks were observed. By analyzing the microstructure and corresponding EDS results (Figure S1, Table S1), the enrichment of Ti in perovskite would result in a relatively lower Ca and Ti and higher Si and Al content in the glassy matrix, which was inferred to possess a higher reactivity in geopolymerization.

**Figure 2:**
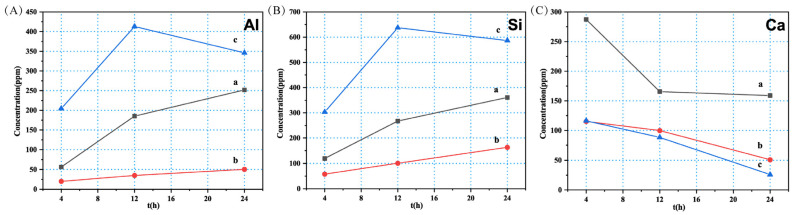
The dissolution of (**A**) Al, (**B**) Si, and (**C**) Ca from modified slags: (a) S–1400 °C–1.0 h; (b) S–N; and (c) S–W during alkali activation.

The modified slags were used as precursors for geopolymerization and subjected to alkali activation. The leaching concentration of metal ions in alkaline solution can be employed as an indicator for evaluating the reactivity of the modified slags [19,20]. Figure 2 demonstrated the leaching result of different modified slags in alkaline solution; the leaching concentration of Al and Si were the highest in S–W and the lowest in S–N. Compared with crystalline phases, the three-dimensional networks of amorphous phase possess lower binding energy for Al–O and Si–O bonds [21], rendering them more susceptible to dissolution under alkaline attack. This suggests that the reactivity of slag in geopolymerization is positively correlated with the amorphous fraction possessing cementitious properties. Considering the XRD analysis and dissolution results, the reactivity of modified slags follows the order of S–W > S–1400 °C–1.0 h > S–N.

Additionally, it was observed that the alkali activation resulted in the dissolution of Ca, which is an inherent element presented in TBBFS. The prolongation of alkali activation did not benefit Ca dissolution; on the contrary, Ca concentration decreased. Ca was either precipitated as Ca(OH)_2_ or converted into C–A–S–H/C–S–H gel by reacting with the dissolved silicate and aluminate species [21]. Under a high concentration of alkali activator, it is plausible that leached Ca^2+^ reacts with OH^-^ to form Ca(OH)_2_ precipitation.

### 2.2. The Structural Properties and Morphology of Porous Geopolymer

**Figure 3:**
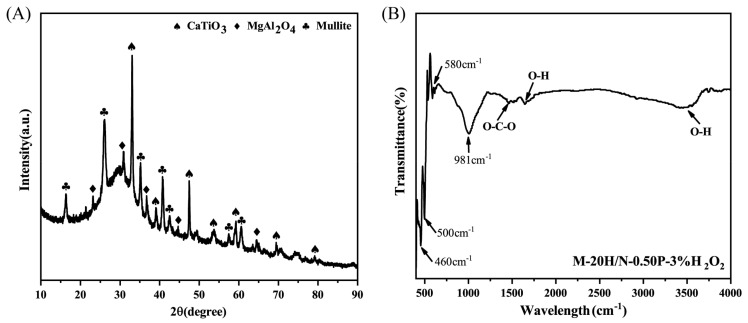
(**A**) XRD pattern of the geopolymer M-20H/N-0.50P-3%H_2_O_2_ and (**B**) FTIR spectra of the geopolymer M-20H/N-0.50P-3%H_2_O_2_.

As verified in Figure S1, the modified slag of S–1400 °C–1.0 h is consisted of crystallized CaTiO_3_ and a glassy matrix. The structure of geopolymer M-20H/N-0.50P-3%H_2_O_2_ was corroborated through powder XRD analysis. As shown in Figure 3A, typical peaks of crystalline phases CaTiO_3_, MgAl_2_O_4_, and mullite were detected, and the latter originated from thermally activated kaolin (Figure S2). The retention of CaTiO_3_ confirmed its non-cementitious nature during geopolymerization. A broad diffraction hump near 30°, which is a characteristic feature of the amorphous phase, indicated structural changes that occurred when the raw materials reacted to form geopolymeric gel with the incorporation of the alkali solution [22,23].

FTIR spectra of the geopolymer M-20H/N-0.50P-3%H_2_O_2_ was displayed in Figure 3B. The signal at 460 cm^−1^ and 500 cm^−1^ can be related to Al–O/Si–O. A clear stretching and bending vibration of Ca–O–Ti at 580 cm^−1^ indicates the presence of CaTiO_3_. A band located at 981 cm^−1^ was attributed to the bending vibration of Si–O–Al or the stretching vibration of Ti–O–Si. Meanwhile, the elongation of O–H bonds can be associated with the signals at 3450 and 1630 cm^−1^, which can be attributed to the presence of water molecules in the material [24]. The signal at 1462 cm^−1^ is the tensile vibration of O–C–O [25], mainly due to the interaction between the particle surface and atmospheric CO_2_.

**Figure 4:**
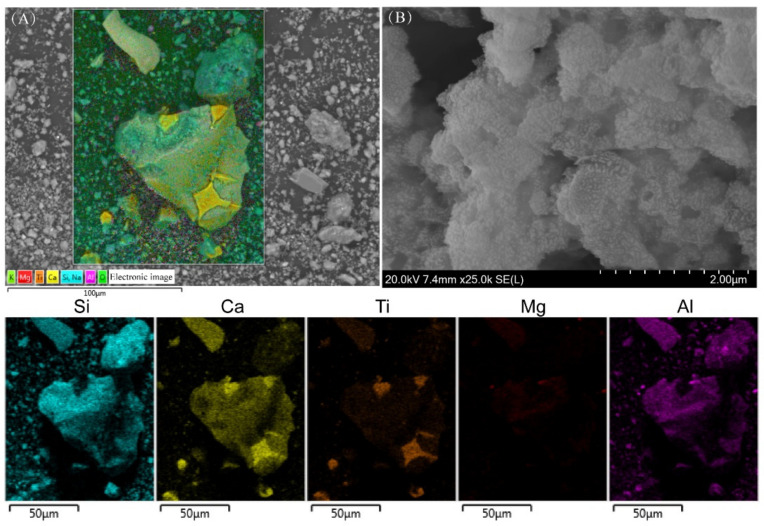
(**A**) EDS surface scanning analysis of the geopolymer M-20H/N-0.50P-3%H_2_O_2_ (Si, Ca, Ti, Mg, and Al elements EDS images were presented at the bottom five subfigures) and (**B**) SEM image of the geopolymer M-20H/N-0.50P-3%H_2_O_2_.

The particle morphology of geopolymer M-20H/N-0.50P-3%H_2_O_2_ was investigated by SEM. In Figure 4A, the 70 μm-sized particles were undissolved and likely originated from TBBFS. The elemental mapping results suggested the rhomboid grains embedded in the undissolved residual particle were rich in Ca and Ti elements, which can be assigned to CaTiO_3_ crystals. The irregular particles in Figure 4B exhibit amorphous assembly, where nano-scale particles are observed at the surface. These formed gels, or nano-crystals, could act as binder phases and bond with undissolved particles [26].

UV–Vis–DRS was measured to describe the photo-adsorption behavior (Figure S3A). Synthetic CaTiO_3_ exhibited strong ultraviolet light absorption with a wide bandgap energy of 3.28 eV (Figure S3B). Slag S–1400 °C–1.0 h and geopolymer M-20H/N-0.5P-3%H_2_O_2_ exhibited a slight increase in the visible light absorption, as indicated by narrower bandgap energies [27]. These results may be due to the red shift resulting from the effect of transition metal ions and nonmetal elements in the samples [7]. Additionally, in the subsequent photocatalytic degradation of MB, due to the synergistic effect of CaTiO_3_ and OH^−^, the actual photocatalytic degradation performance was significantly better than CaTiO_3_ and modified slag. The details are discussed later in Section 2.4.

The N_2_ adsorption–desorption curves were adopted to determine the textural properties. As shown in Figure S4, the prepared samples presented a Type IV isotherm with a Type H3 hysteresis loop, which indicates a slit-shaped mesoporous structure. Table 1 shows that the specific surface area (S_BET_), average pore size, and pore volume are in the range of 52.24~91.52 m^2^/g, 9.22~12.06 nm, and 0.143~0.253 cm^3^/g, respectively. The addition of H_2_O_2_ can increase the specific surface area of geopolymers. The specific surface areas of the geopolymers prepared under 1 wt.%, 3 wt.%, and 5 wt.% H_2_O_2_ were 56.12 m^2^/g, 60.74 m^2^/g, and 72.33 m^2^/g, respectively. Meanwhile, the n(H_2_O)/n(Na) had little effect on the specific surface area. When increasing from 12 to 20, the specific surface area of the geopolymer only increased from 52.24 m^2^/g to 80.32 m^2^/g. Due to the lower activity of modified slag compared to metakaolin and fumed silica, when a high volume of TBBFS was introduced as a precursor in alkaline activation, the specific surface area of the resulting geopolymer decreased. According to the previous analysis of the reactivity of different slag precursors, the prepared geopolymer-specific surface area obtained consistent results. Still, geopolymer M-20H/N-0.50P-3%H_2_O_2_ exhibited a high BET surface area of 80.32 m^2^/g.

### 2.3. The Effect of Processing Parameters on the Adsorption-Photocatalytic Degradation Behavior

**Figure 5:**
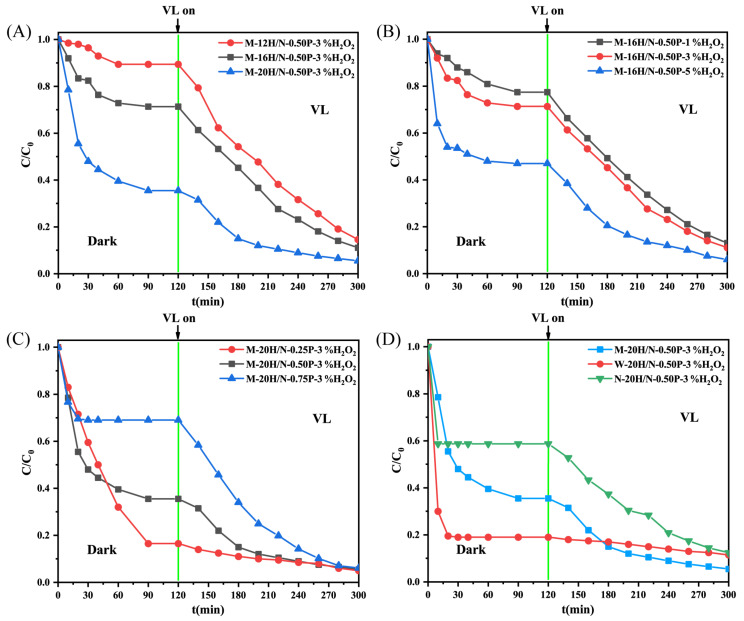
Dark adsorption and visible light photocatalysis of geopolymer synthesized under different conditions: (**A**) n(H_2_O)/n(Na): 12, 16, and 20; (**B**) H_2_O_2_ addition: 1 wt.%, 3 wt.%, and 5 wt.%; (**C**) modified slag proportion: 0.25, 0.50, and 0.75 and (**D**) the type of modified slags used: S–1400 °C–1.0 h, S–W, and S–N (40 °C, 50 mg of dosage).

The MB removal tests were performed independently in the dark and under a visible light source. The difference in the MB removal efficiency between different geopolymers was shown in Figure 5. The adsorption–desorption equilibrium can be well-established in 120 min, independent of various conditions. The adsorption towards MB removal benefited from higher n(H_2_O)/n(Na), the addition of H_2_O_2_, and the low proportional use of modified slag. The order of adsorption capacity of geopolymer is successively W-20H/N-0.50P-3%H_2_O_2_ > M-20H/N-0.50P-3%H_2_O_2_ > N-20H/N-0.50P-3%H_2_O_2_. It implies that higher S_BET_ with more available sites favors MB adsorption. As for the photocatalytic activity, it will be discussed later in Section 2.3.4.

#### 2.3.1. Adsorption Kinetic Characteristics

**Figure 6:**
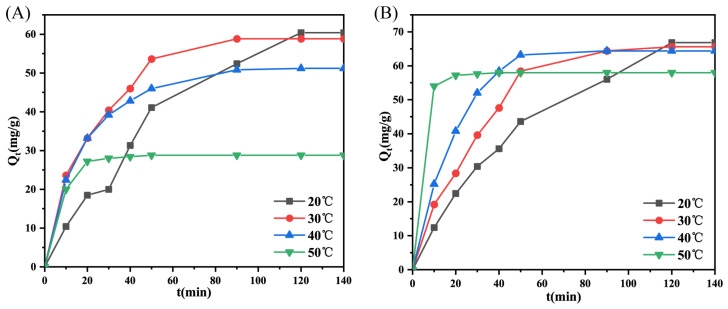
Adsorption kinetics curves of geopolymers: (**A**) M-20H/N-0.50P-3%H_2_O_2_ and (**B**) W-20H/N-0.50P-3%H_2_O_2_ at different temperatures.

**Figure 7:**
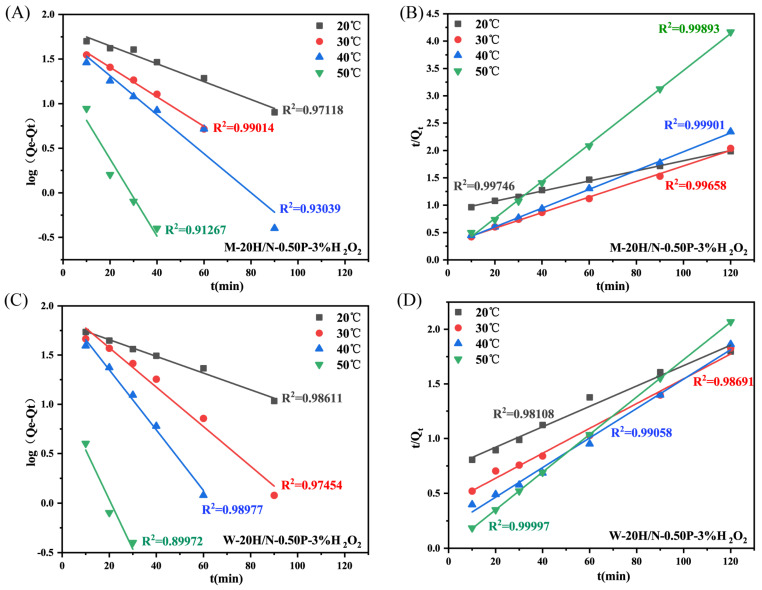
Fitting plots of the (**A**,**C**) pseudo–first–order kinetics and (**B**,**D**) pseudo–second–order kinetics of MB adsorption on geopolymers.

The geopolymers derived from modified slags, i.e., S–1400 °C–1.0 h, S–W were selected to study their adsorption kinetics, which helps us to comprehend the underlying interactions between adsorbents and adsorbates. Figure 6 displays the kinetic curves of geopolymer M-20H/N-0.50P-3%H_2_O_2_ and W-20H/N-0.50P-3%H_2_O_2_ at different temperatures. It was found that temperature increments did not enhance adsorption capacity but shortened the time to attain equilibrium, suggesting the adsorption is exothermic [28]. The equations of the models were presented in the Supplemental Materials. The fitting results in Figure 7 show that the pseudo-second-order model presents the highest correlation coefficient, which signifies that the chemical interactions between MB molecules and the geopolymer surface are the rate-limiting step [29].

#### 2.3.2. Adsorption Thermodynamics

Adsorption thermodynamic parameters, i.e., equilibrium constant (*K_C_*, L/mg), Gibb’s free energy change (Δ*G*, KJ/mol), and enthalpy change (Δ*H*, KJ/mol), as well as entropy change (Δ*S*, KJ/(K·mol)), were assessed to evaluate the feasibility, nature, and favorability of adsorption.

(1) $$K_{C}=\frac{Q_{e}}{C_{e}}\;$$

(2) $$\Delta G=-RT\ln K_{C}$$

(3) $$\ln K_{c}=-\frac{\Delta H}{RT}+\frac{\Delta S}{R}$$

In Equations (1)–(3) [30]: *Q_e_*: the amount of MB dye per unit mass of absorbent at equilibrium, mg/g; *C_e_:* equilibrium concentration of MB in solution, mg/L; *R*: ideal gas constant, 8.314 J/(mol·K); *T*: thermodynamic temperature, K.

The results for adsorption thermodynamics are presented in Table 2 and Figure S5. The adsorption process of geopolymers onto MB is exothermic, as indicated by a negative value of enthalpy change Δ*H*. The negative Δ*G* value indicates that the adsorption is a thermodynamic spontaneous process. As the temperature increases, its value rises, while the corresponding equilibrium constant decreases, signifying that higher temperature is unfavorable for adsorption.

#### 2.3.3. Adsorption Isotherms

**Figure 8:**
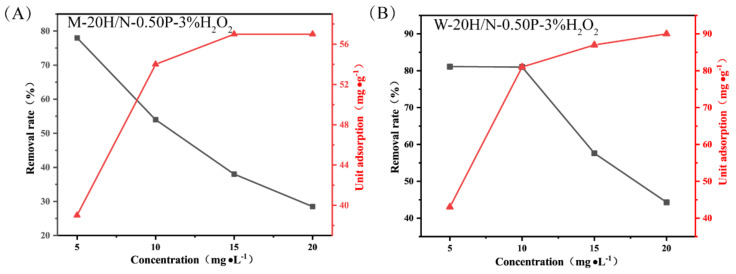
The removal performance curves for MB with various initial concentrations of (**A**) M-20H/N-0.50P-3%H_2_O_2_ and (**B**) W-20H/N-0.50P-3%H_2_O_2_.

The adsorption isotherms were conducted under the conditions of 20 °C, 120 min, and 20 mg of adsorbent to investigate homogeneous or heterogeneous adsorption behavior. As seen in Figure 8, the *Q_e_* values for geopolymer M-20H/N-0.50P-3%H_2_O_2_ and W-20H/N-0.50P-3%H_2_O_2_ increase with rising initial concentration, while the removal rate of MB decreases. The Langmuir model [31], Freundlich model [32], and Temkin model [33] (represented by the L model, F model, and T model) were adopted to fit the adsorption isotherms, with equations detailed in the Supplement Materials.

As illustrated in Figure S6 and Table 3, the fitting results indicate that the adsorption isotherms are well-described by the Langmuir model, suggesting that the adsorption is monolayer adsorption. The maximum adsorption capacity (*Q*_0_, mg/L) calculated using the L model is 59.31 mg/g at 20 °C for geopolymer M-20H/N-0.50P-3%H_2_O_2_, and 97.18 mg/g for geopolymer W-20H/N-0.50P-3%H_2_O_2_.

Furthermore, a direct comparison of MB adsorption capacities with S_BET_ among different materials is conducted. The results in Table 4 indicate that the MB adsorption capacity is strongly correlated with the specific surface area of the materials. Moroccan clay-based geopolymer with a high S_BET_ of 128 m^2^/g exhibited an adsorption capacity of 183 mg/g [34]. Studies by Novais et al. [35] verified that the adsorption capacity of geopolymer can be enhanced through a thermal regeneration process. The adsorption capacity of fly ash geopolymer spheres increased from 30.1 mg/g (1st) to 79.9 mg/g (9th) [36]. Compared to other reported materials, the as-prepared geopolymers from TBBFS possess high S_BET_ and excellent adsorption performance for MB removal.

#### 2.3.4. Photodegradation Kinetics

**Figure 9:**
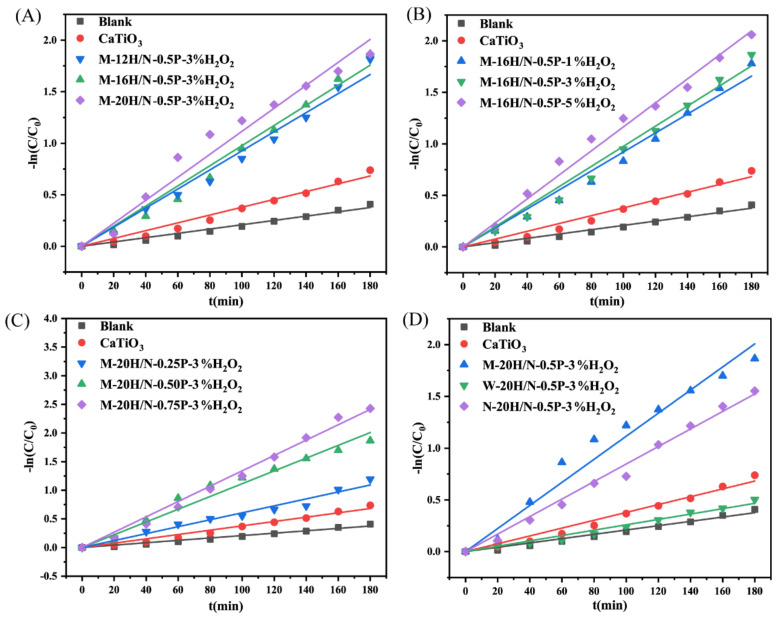
(**A**–**D**) Photocatalytic degradation (−ln(*C*/*C*_0_) against light irradiation time) of MB at 40 °C of geopolymer with a dosage of 50 mg.

To assess the photocatalytic activities of the prepared geopolymers, synthesized CaTiO_3_ was selected for comparison. As shown in Figure 9A–D, the ln(*C*/*C_e_*) plotted against light irradiation time exhibits a linear relationship, revealing that degradation curves of MB follow pseudo-first-order kinetics, which align with the Langmuir–Hinshelwood (L–H) model when the solution is diluted (<1 × 10^−3^) [37,38,39].

(4) $$-\ln\left(\frac{C}{C_{e}}\right)=k_{ap}t$$

where *C* is the concentration of MB at time interval *t* under visible light illumination (mg/L), *k_ap_* is the apparent rate constant of a pseudo-first-order reaction (min^−1^).

The self-decomposition of MB under visible light irradiation (blank: without catalyst) is negligible. Compared to that of pure CaTiO_3_, the degradation efficiencies for MB substantially improve when geopolymer material is added to MB solution, except for material W-16H/N-0.50P-3%H_2_O_2_. Figure 9A–C reveal that processing parameters, i.e.*,* n(H_2_O)/n(Na), the addition of H_2_O_2_, and the use of modified slag, positively affect the enhancement of photocatalytic activity.

As seen in Table 5, a comparison of MB removal performance and apparent rate constant *k_ap_*, among various materials, was made. CaTiO_3_ typically possesses a large bandgap energy and exhibits photocatalytic activity under ultraviolet light irradiation. By combining Ca(OH)_2_, the CaTiO_3_/Ca(OH)_2_ composite exhibits a superior photocatalytic activity with a *k_ap_* value of 0.2168 min^−1^ under ultraviolet light irradiation [40]. However, under visible light irradiation, even after HNO_3_ acidification, modified CaTiO_3_ exihibits a slightly higher *k_ap_* value of 0.00359 min^−1^ compared to unmodified CaTiO_3_ [41]. Considering the adsorption–photocatalytic degradation performance in Figure S7 and Table 5, the obtained geopolymers were demonstrated to be promising candidates for dye removal. The *k_ap_* values of modified slag of S–1400 °C–1.0 h and S–N are comparable to that of pure CaTiO_3_. Geopolymer M-20H/N-0.5P-3%H_2_O_2_ exhibits enhanced photocatalytic activity with a considerably higher *k_ap_* value of 0.01115 min^−1^, implying that the visible light photocatalytic efficiency for MB reaches 93.5% in 180 min with a geopolymer dosage of 50 mg.

### 2.4. Possible Interaction Mechanism

**Figure 10:**
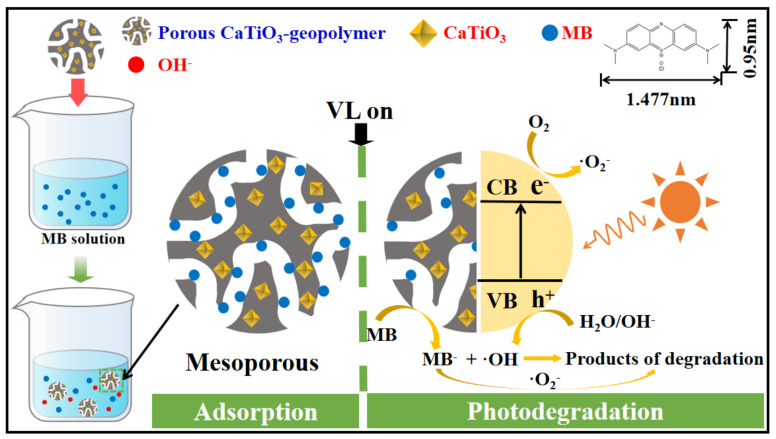
Possible interactions between MB and porous CaTiO_3_–geopolymer.

The possible interaction between porous geopolymer and MB is illustrated in Figure 10. The adsorptive–photocatalytic process of MB removal comprises successive steps: (i) The mass transfer of MB molecules from the liquid phase to the geopolymer surface via external and intraparticle diffusion; (ii) The chemisorption of MB on the surface via surface interactions; and (iii) The photodegradation of MB by CaTiO_3_. As a cationic dye, the adsorption of MB molecules is facilitated by the negatively charged surface of adsorbents [44,45]. Table 6 illustrates the pH changes of the MB solution during the adsorption–photodegradation process. After the addition of geopolymer powders to the MB solution, the pH value of the slurry increased to approximately 10 due to the leaching of OH^−^. The geopolymer can act as a pH buffer by releasing hydroxyl ions in the waste treatment system [46]. This suggested that the cationic MB^+^ can absorb easily onto the geopolymer because of electrostatic attraction, forming hydrogen bonds between a hydroxyl group (-OH) and a nitrogen atom in the MB molecule [44].

When the illuminated light energy is larger than the bandgap of CaTiO_3_, CaTiO_3_ absorbs the light energy to form photogenerated electron-hole pairs (Equation (5)) at the surface of particles. To prevent the recombination of electron-hole pairs, they have to participate in the redox reactions by combining with the oxidant (Equations (6) and (7)) and reductant (Equation (8)), respectively. According to the pH variation in Table 6, its value gradually decreased, along with the photodegradation process. This indicated that the OH^-^ released by geopolymer was consumed by photogenerated holes to generate strong oxidative ·OH radicals.

(5) $${\mathrm{CaTiO}}_{3}+\mathrm{hv}\to e^{-}+h^{+}$$

(6) $$e^{-}+O_{2}\to O_{2}{∙}^{-}$$

(7) $$O_{2}{∙}^{-}+H_{2}O\to \mathrm{OH}+{\mathrm{HO}}_{2}∙+H_{2}O_{2}$$

(8) $$h^{+}+H_{2}O/{\mathrm{OH}}^{-}\to ∙\mathrm{OH}+H^{+}$$

(9) $$h^{+}\mathrm{or}∙\mathrm{OH}+\mathrm{MB}\to \mathrm{degradation}\;\mathrm{products}$$

Hence, due to the synergistic effects between CaTiO_3_ and geopolymer, the coupling of CaTiO_3_ with geopolymer material possesses the following advantages: (i) The large specific surface area and mesoporous structure benefit the inward penetration of MB solution via pore channels, and more active sites are exposed for adsorption and photodegradation; (ii) Compared to solely photocatalytic material, the buffering of OH^−^ not only favors the adsorption via electrostatic attraction but also promotes the oxidation of MB dye by enhancing the formation of hydroxyl radicals [40].

## 3. Materials and Methods

### 3.1. Raw Materials

The TBBFS, kaolin, fumed silica (SiO_2_ > 98.0 wt.%), sodium hydroxide (NaOH), cetyltrimethylammonium bromide (CTAB), hydrogen peroxide (H_2_O_2_), and methylene blue (MB) were used as raw materials and reagents. The chemical compositions of TBBFS and kaolin were measured by X-ray fluorescence (XRF, Panalytical Axios, Holland); the results are shown in Table 7. The as-received TBBFS has a relatively high content of TiO_2_ and a basicity (mass ratio of CaO/SiO_2_) of 1.04. Considering its low available Si and Al content, the supplementary Si and Al sources, such as fumed silica and kaolin, were incorporated during the geopolymerization. Thermally activated kaolin was prepared by heating kaolin at 750 °C for 3 h.

### 3.2. Experimental Methods

**Figure 11:**
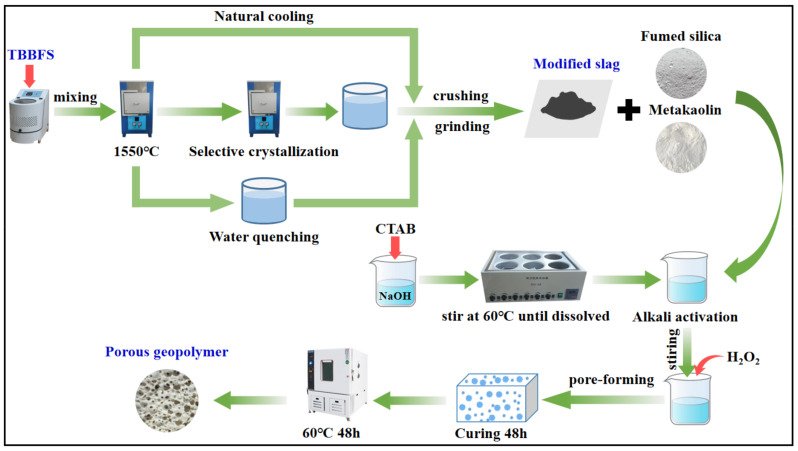
Schematic diagram illustrating the synthetic route of porous geopolymer.

Figure 11 schematically illustrates the synthetic route of porous geopolymer. The process consisted of steps involved in thermal modifications, alkali activation, pore-forming, and curing.

#### 3.2.1. Thermal Treatment of TBBFS

TBBFS was ball milled (XQM-1L, Tianchuang, Changsha, China) and then placed in a tube furnace for heating to a molten state (1550 °C). After being completely melted, the molten slag was transferred to a preheated furnace to initiate the crystallization of the perovskite phase for a duration at the desired temperature. Followed by this, water quenching was performed. The obtained slag was crushed and ground into fine powders (<0.15 mm). The modified slag of S–1400 °C–1.0 h represented TBBFS underwent selective crystallization at the conditions of 1400 °C, 1.0 h of duration. In addition to selective crystallization, natural cooling and water quenching were adopted. Water-quenched slag (S-W) was prepared by directly pouring the molten slag into water (15 ± 5 °C). Natural-cooled slag (S–N) was prepared by cooling the molten slag in a furnace at a relatively slow rate (7–8 h).

#### 3.2.2. Synthesis of Porous Geopolymer

The porous geopolymers were synthesized via alkali activation, pore-forming, and curing. The mole ratio of Si:Al:Na in the precursor was adjusted to 2:1:1. As presented in Table 8, the effects of slag proportion, the mole ratio of H_2_O/Na, H_2_O_2_ addition amount, and raw material were studied. In the process of alkali activation, 3 wt.% CTAB was added into the NaOH solution and stirred in a water bath until completely dissolved. Then, modified slag powders, fumed silica, and metakaolin were added into the solution and were mechanically agitated for 10 min to ensure a uniform slurry. A certain amount of H_2_O_2_ was added to the slurry drop-wise, along with continuous stirring, for pore formation. The slurry was cast in an 8 × 8 × 8 cm^3^ plastic mold and cured in a sealed bag at room temperature for 48 h, and then another 48 h in an oven at 60 °C. After curing, the porous geopolymer was ground into powders for characterization and tests.

#### 3.2.3. Synthesis of Pure CaTiO_3_

Powder mixtures were prepared by weighing, and mixing CaO and TiO_2_ chemicals based on the stoichiometric ratio of CaTiO_3_. The mixtures were dried and then pressed into pellets under a pressure of 30 MPa. The pellets were put into a tube furnace and calcined at 1400 °C for 3 h to synthesize pure CaTiO_3_.

### 3.3. Adsorption and Photocatalytic Degradation Tests

MB solution of 200 mL, with a concentration of 20 mg/L, was used as a representative organic dye to evaluate the adsorption and photocatalytic properties of the porous geopolymers. The adsorption tests were performed in a glass reactor equipped with a magnetic stir and a thermostat in the dark. Sampling was conducted at certain intervals. Table S2 shows the summary of the experimental design for adsorption tests. The adsorption can be regarded as being in equilibrium once the MB concentration no longer changed. Then, the photocatalytic degradation tests were carried out under visible light irradiation with a 300 W Xenon lamp (PLS-SXE300, Perfect Light, Beijing, China). The sampling was conducted with a time interval of 10 min, and the suspension was separated by centrifugation. The residual MB concentration was measured with an ultraviolet-visible (UV-Vis) spectrophotometer (TU-1810, Puxi, Beijing, China) at a wavelength of 660 nm.

The adsorption capacity toward MB is measured from the following Equation (10):

(10) $$Q_{t}=\frac{\left(C_{0}-C_{t}\right)\times V}{m}\;$$

where *Q_t_* is the amount of MB dye uptake per unit mass of absorbent at time *t*, mg/g; *C*_0_ and *C_t_* represent the initial and residual concentrations of MB after time *t*, respectively, mg/L; *V* represents the volume of the solution, mL; *m* is the mass of the adsorbent, mg.

### 3.4. Characterization

The reactivity of modified slags in terms of Si, Al, and Ca dissolution was characterized by immersing slag powders in a 6 mol/L NaOH solution with a solid-to-liquid mass ratio of 1:40 at room temperature. The dissolved Si, Al, and Ca concentrations in the leachate were measured through the inductively coupled plasma–atomic emission spectrometry (ICP-AES) technique (Agilent 5100, Agilent Technologies, Los Angeles, CA, USA).

X-ray diffraction (XRD, Ultima IV, Rigaku, Tokyo, Japan, Cu radiation) was performed to determine the crystalline phases at a scanning rate of 20°/min from 10° to 90°. The scanning electron microscope (SEM, SU-5000, HITACHI, Tokyo, Japan) and energy dispersive spectrometer (EDS, X-MAX, Oxford, Oxford, UK) was used to observe the morphologies and analyzed the contents of elements in the micro-areas. The Brunauer–Emmett–Teller (BET, TriStar II 3020, Micromeritics, GA, USA) surface aera was analyzed using N_2_ as the adsorption gas, degassed at 300 °C for 8 h. In addition, the mesopore size distribution was calculated from the adsorption branch of isotherm by the Barrett–Joyner–Halenda (BJH) model. The UV–Vis diffuse reflectance spectrum (UV–Vis–DRS, Lambda 950, PerkinElmer, MA, USA) was measured using BaSO_4_ as reference material. Fourier transform infrared spectrometer (FTIR, Nicolet-is 10, Thermo Fisher, MA, USA) was measured to obtain chemical bonds and function group information.

## 4. Conclusions

The utilization of metallurgical wastes and effective activation as porous geopolymers is of great significance. In this work, TBBFS was thermally modified and then utilized as a precursor for porous geopolymers. The properties and performance of the resulting geopolymer in terms of adsorptive–photocatalytic activity towards MB removal can be greatly varied by the processing parameters. The results enable the following conclusions to be reached.

1. TBBFS subjected to water quenching (fast cooling) was amorphous and resulted in the highest S_BET_ in the derived geopolymer, which exhibited the highest MB adsorption capacity of 97.18 mg/g.
2. The adsorption process could be explained by a pseudo-second-order kinetic model and a Langmuir adsorption isotherm model. The adsorption results showed that the removal of MB was a spontaneous, exothermic, monolayer chemisorption process. Therefore, it was significantly affected by the specific surface area of the adsorbent.
3. Selective crystallization of TBBFS at 1400 °C for 1 h gave the resulting geopolymer photocatalytic activity. The release of OH^-^ from geopolymer promotes both adsorption and photocatalytic degradation performances. The maximum removal rate for MB can reach 93.5% in 180 min. The n(H_2_O)/n(Na), the addition of H_2_O_2_, and the use of modified slag had a positive effect on the improvement of photocatalytic activity.

The present study demonstrated that the properties and performances of geopolymers can be optimized by controlling slag chemistry. Porous geopolymer prepared from TBBFS had excellent adsorption and photodegradation performance. It provides a new way for the utilization of TBBFS in environmental purification under visible light.

## Figures and Tables

**Table 1:** Textural properties of porous geopolymers.

Material	S_BET_ (m^2^/g)	Pore Volume (cm^3^/g)	Average Pore Size (nm)
M-12H/N-0.50P-3%H_2_O_2_	52.24	0.143	9.75
M-16H/N-0.50P-1%H_2_O_2_	56.12	0.208	11.32
M-16H/N-0.50P-3%H_2_O_2_	60.74	0.202	11.79
M-16H/N-0.50P-5%H_2_O_2_	72.33	0.191	10.16
M-20H/N-0.50P-3%H_2_O_2_	80.32	0.227	10.93
M-20H/N-0.25P-3%H_2_O_2_	87.58	0.253	11.31
M-20H/N-0.75P-3%H_2_O_2_	62.44	0.184	9.97
W-20H/N-0.50P-3%H_2_O_2_	91.52	0.219	9.22
N-20H/N-0.50P-3%H_2_O_2_	66.27	0.217	12.06

**Table 2:** Adsorption thermodynamic parameters of geopolymers.

Material	*T* (K)	*K_C_*	Δ*G* (kJ/mol)	Δ*H* (kJ/mol)	Δ*S* (kJ/K·mol)
M-20H/N-0.50P-3%H_2_O_2_	293	21.000	−7.416	−56.37328	−0.16678
	303	10.316	−5.879		
	313	5.412	−4.394		
	323	2.381	−2.330		
W-20H/N-0.50P-3%H_2_O_2_	293	19.647	−7.254	−7.94761	−0.00234
	303	17.730	−7.243		
	313	16.100	−7.231		
	323	14.483	−7.178		

**Table 3:** Isotherms parameters for MB adsorption on geopolymers.

Isotherm	Parameters	M-20H/N-0.50P-3%H_2_O_2_	W-20H/N-0.50P-3%H_2_O_2_
L model	Q_0_ (mg/g)	59.31	97.18
	K_L_ (L/mg)	2.01	1.22
	R^2^	0.99956	0.99114
F model	n	3.87	6.45
	K_F_ (L/g)	52.59	39.74
	R^2^	0.50760	0.86593
T model	B (J/mole)	7.35	16.66
	A (L/g)	224.18	26.77
	R^2^	0.88322	0.57132

**Table 4:** Comparison of adsorption capacity of various absorbents for MB removal.

Adsorbents	Temperature (°C)	BET Surface Area (m^2^/g)	Adsorption Capacity (mg/g)	Ref.
Phosphoric acid-based geopolymer	28	2.28	3.01	[33]
Moroccan clay-based geopolymer	-	128	183	[34]
Biomass fly ash-geopolymer monoliths	-	-	15.4 (1st), 20.5 (2nd), 20.4 (3rd)	[35]
Fly ash geopolymer balls	Ambient temperature	-	30.1 (1st), 79.7 (9th)	[36]
M-20H/N-0.50P-3%H_2_O_2_	20	72.33	59.31	This work
W-20H/N-0.50P-3%H_2_O_2_	20	91.52	97.18	This work

**Table 5:** Comparison of photocatalytic performance of various materials for MB removal.

Photocatalyst	Catalyst Dose (mg) vs. vol (mL) and MB Conc (mg/L)	Performance	*k_ap_* (min^−1^)	Ref.
CaTiO_3_/Ca(OH)_2_	100 vs. 100 of 10^−5^ mol/L	82.3% degradation in 8 min	0.2168	[40]
CaTiO_3_	100 vs. 200 of 10	44.29% degradation in 325 min	0.0018	[41]
HNO_3_ acidified CaTiO_3_ (pH = 2)	100 vs. 200 of 10	66.86% degradation in 325 min	0.00359	[41]
Graphite oxide grafted titanate nanotubes	20 vs. 100 of 20	97.5% degradation in 120 min (30 min adsorption and 90 min UV irradiation)	0.02845	[42]
CuO@calcium silicate hydrate	20 vs. 50 of 20	90% degradation in 150 mi	0.0119	[43]
CaTiO_3_	50 vs. 200 of 20	52.24% degradation in 180 min	0.00379	This work
S-N	50 vs. 200 of 20	57.63% degradation in 180 min	0.00437	This work
S-1400 °C–1.0 h	50 vs. 200 of 20	54.41% degradation in 180 min	0.00390	This work
N-20H/N-0.50P-3%H_2_O_2_	50 vs. 200 of 20	87.56% degradation in 180 min	0.0084	This work
M-20H/N-0.50P-3%H_2_O_2_	50 vs. 200 of 20	93.5% degradation in 180 min	0.01115	This work

**Table 6:** The pH changes during the removal process of MB by geopolymers.

	pH Initial	pH Adsorption Equilibrium	Photodegradation Time (min)						
			20	40	60	80	100	140	180
M-12H/N-0.50P-3%H_2_O_2_	10.02	10.02	9.84	9.61	9.47	9.29	9.11	8.71	8.28
M-16H/N-0.50P-3%H_2_O_2_	9.97	9.97	9.78	9.60	9.46	9.26	9.09	8.67	8.25
M-20H/N-0.50P-3%H_2_O_2_	9.93	9.93	9.75	9.59	9.45	9.24	9.05	8.63	8.21

**Table 7:** Chemical composition (wt.%) of TBBFS and kaolin.

Component	CaO	SiO_2_	MgO	Al_2_O_3_	TiO_2_	Fe_2_O_3_	MnO
TBBFS	26.13	25.01	8.76	12.94	24.42	2.00	0.65
Kaolin	1.28	52.51	0.12	45.50	0.00	0.59	0.00

**Table 8:** Process parameters for the porous geopolymers synthesis.

Material	Raw Material	Slag Proportion (wt.%)	n(H_2_O)/n(Na)	H_2_O_2_ Addition Amount (wt. %)
M-12H/N-0.50P-3%H_2_O_2_	S–1400 °C–1.0 h	0.50	12	3
M-16H/N-0.50P-1%H_2_O_2_	S–1400 °C–1.0 h	0.50	16	1
M-16H/N-0.50P-3%H_2_O_2_	S–1400 °C–1.0 h	0.50	16	3
M-16H/N-0.50P-5%H_2_O_2_	S–1400 °C–1.0 h	0.50	16	5
M-20H/N-0.50P-3%H_2_O_2_	S–1400 °C–1.0 h	0.50	20	3
M-20H/N-0.25P-3%H_2_O_2_	S–1400 °C–1.0 h	0.25	20	3
M-20H/N-0.75P-3%H_2_O_2_	S–1400 °C–1.0 h	0.75	20	3
W-20H/N-0.50P-3%H_2_O_2_	S–W	0.50	20	3
N-20H/N-0.50P-3%H_2_O_2_	S–N	0.50	20	3

## Data Availability

Not applicable.

## Supplementary Materials

The following supporting information can be downloaded at: https://www.mdpi.com/article/10.3390/molecules28093673/s1, Figure S1: SEM images of modified slags: (A) S-1400 °C–1.0 h, (B) S-N and (C) S-W. (C: CaTiO_3_; M: MgAl_2_O_4_); Table S1: EDS point scan data in modified slag matrix; Figure S2: XRD pattern of the kaolin; Figure S3: (A) UV-Vis-DRS of the prepared samples and (B) Tauc plots of the corresponding samples; Figure S4: N_2_ adsorption-desorption and pore size distribution: (A) M-20H/N-0.25P-3%H_2_O_2_; (B) W-20H/N-0.25P-3%H_2_O_2_, and (C) N-20H/N-0.25P-3%H_2_O_2_; Figure S5: Adsorption thermodynamics of geopolymer: (A) M-20H/N-0.50P-3%H_2_O_2_ and (B) N-20H/N-0.50P-3%H_2_O_2_; Figure S6: Corresponding adsorption isotherms for MB fitted by L model, F model, and T model of M-20H/N-0.50P-3%H_2_O_2_ and W-20H/N-0.50P-3%H_2_O_2_; Figure S7: (A) Direct adsorption-photodegradation curves under different samples; (B) Photocatalytic degradation (−ln(C/C_0_) against light irradiation time) of MB at 40 °C of samples with a dosage of 50 mg; Table S2: Experimental design for adsorption tests.
