# Supplementary material for: A Porous Geopolymer Containing Ti-Bearing Blast Furnace Slag: Synthesis, Characterization, and Adsorption-Photodegradation Studies towards Methylene Blue Removal under Visible Light Condition
Source: Molecules. 2023 Apr 24;28(9):3673. doi: 10.3390/molecules28093673 (PMC10180165; doi:10.3390/molecules28093673)
Supplement: Supplementary file 1 [file molecules-28-03673-s001.zip › molecules-2326901-SI.pdf]

Supplementary data

A porous geopolymer containing Ti-bearing blast furnace slag: synthesis, characterization, and adsorption-photodegradation studies towards methylene blue removal under visible light condition

Yijian Cheng[1], Kun Wang[2], Peng Li[1,*], Hongwei Guo[1], Bingji Yan[1,3], Dong Chen[1,*], Wei Zhao[1,4]

1 Shagang School of Iron and Steel, Soochow University, Suzhou 215021, China; chengyijian21@163.com (Y.C.)

2 State Key Laboratory of Heavy Oil, China University of Petroleum (East China), Qingdao 266580, China

3 State Key Laboratory of Advanced Metallurgy, University of Science and Technology Beijing, Beijing 100083, China

4 Key Laboratory of Metallurgical Emission Reduction & Resources Recycling (Anhui University of Technology), Ministry of Education, Maanshan 243002, China

(*Corresponding authors: lipeng@suda.edu.cn (P.L.); chend@suda.edu.cn (D.C.)

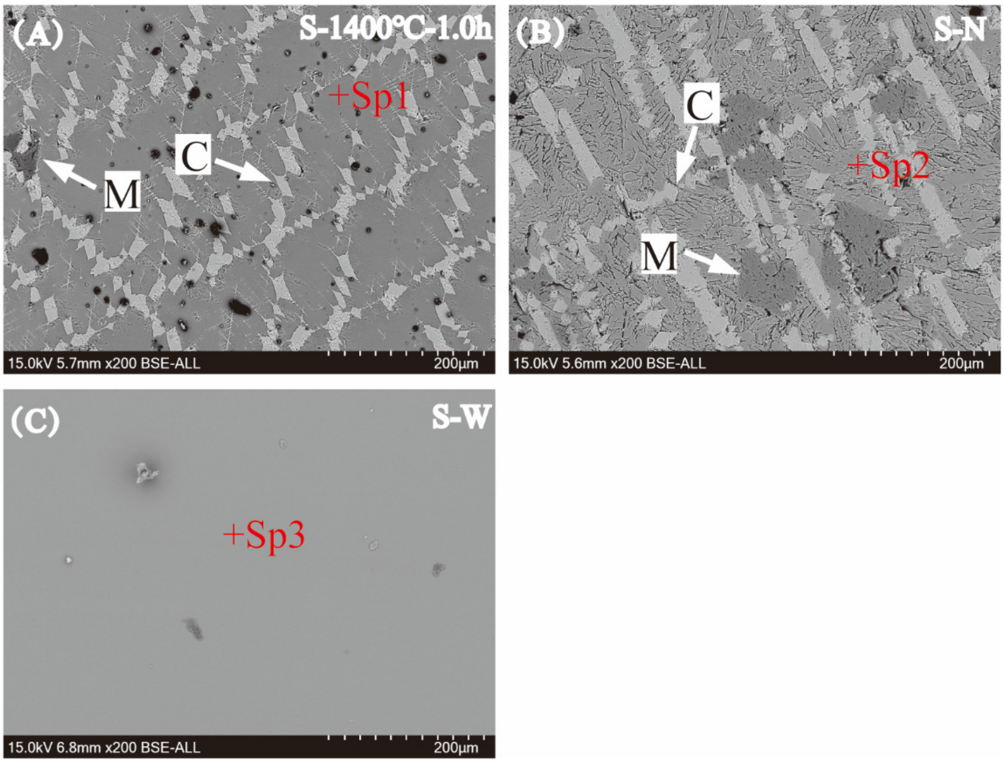

Figure S1. SEM images of modified slags: (A) S-1400°C-1.0h, (B) S-N and (C) S-W. (C: $CaTiO_3$; M: $MgAl_2O_4$).

Table S1. EDS point scan data in modified slag matrix.

		Main element composition (wt.%)					
		O	Ca	Si	Al	Ti	Mg
Sp1	S-1400℃-1.0h	42.7	15.1	15.1	10.6	5.0	5.6
Sp2	S-N	42.2	14.8	14.1	14.0	4.5	5.9
Sp3	S-W	42.7	20.3	10.6	9.3	12.7	4.4

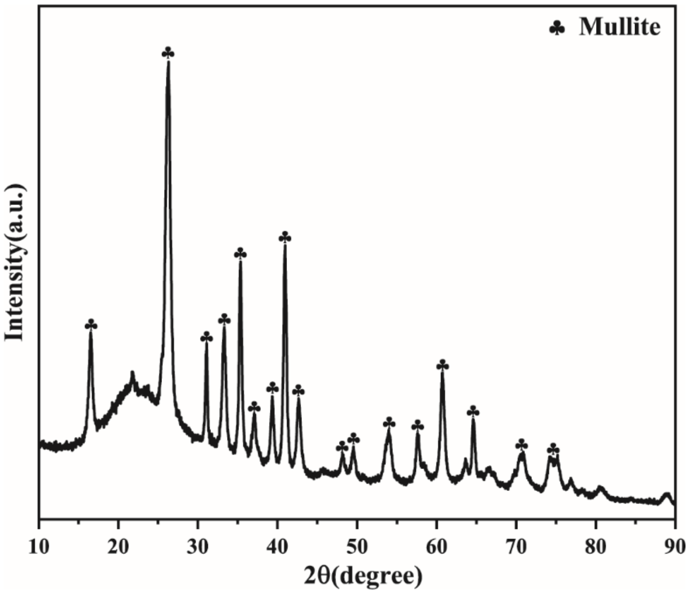

Figure S2. XRD pattern of the kaolin.

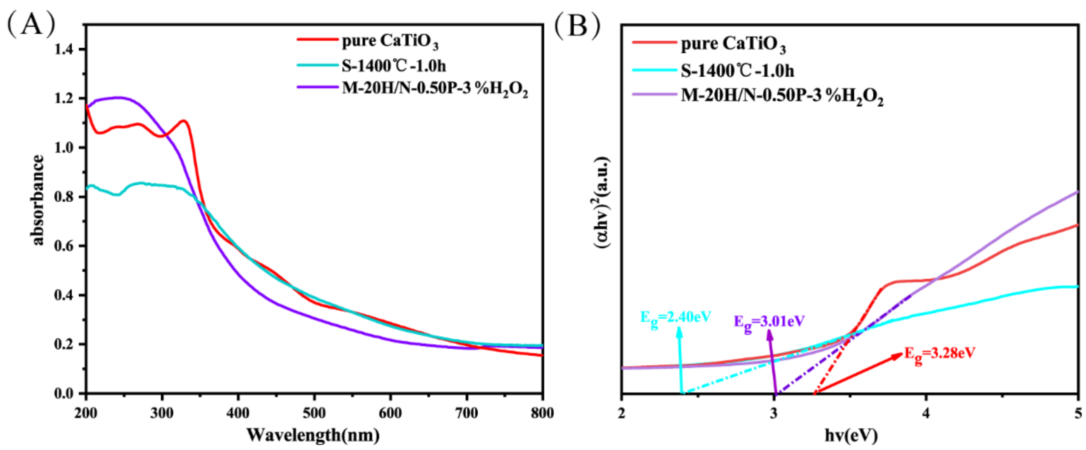

Figure S3. (A) UV-Vis-DRS of the prepared samples and (B) Tauc plots of the corresponding samples.

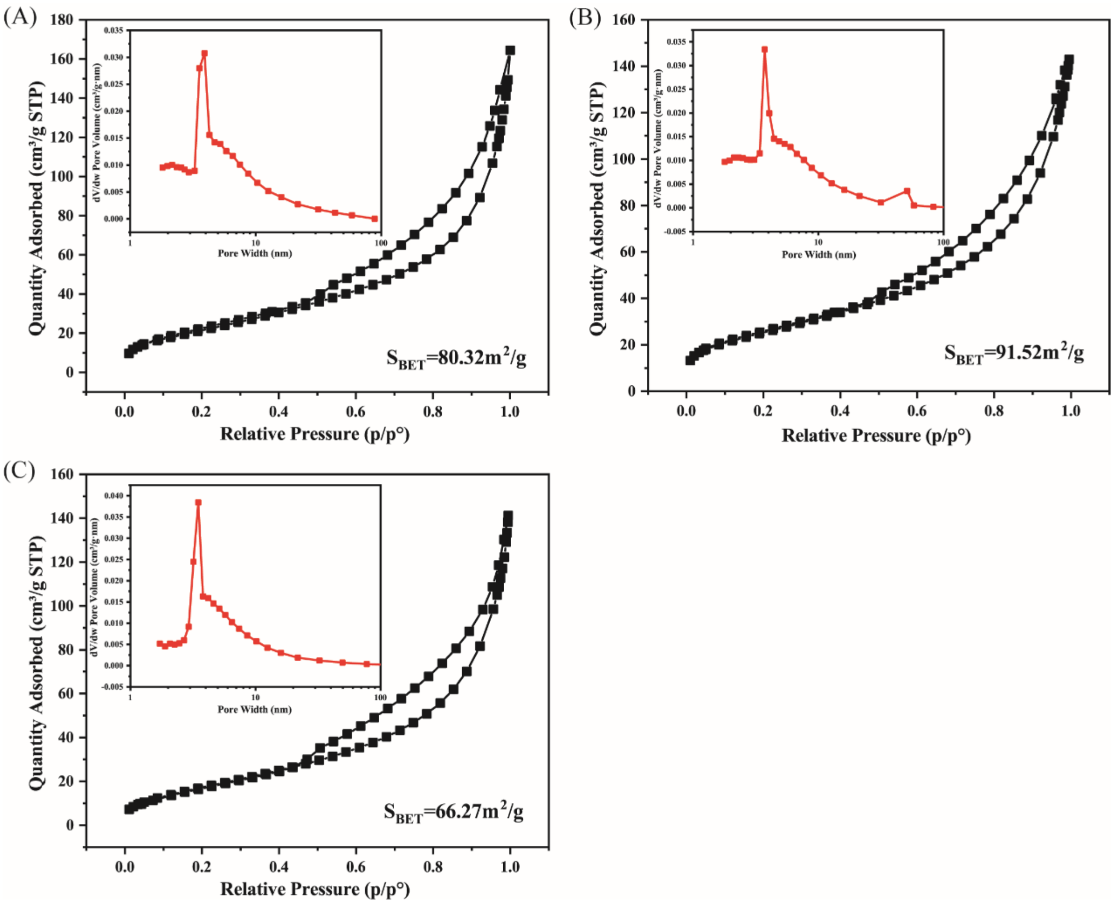

Figure S4. N_2 adsorption-desorption and pore size distribution: (A) M-20H/N-0.25P-3%H_2O_2; (B) W-20H/N-0.25P-3%H_2O_2, and (C) N-20H/N-0.25P-3%H_2O_2.

S2.3.1. Adsorption kinetic characteristics

The adsorption data were fitted to the pseudo-first-order and pseudo-second-order rate equations. Pseudo-first-order rate equation for adsorption in liquid-solid systems described in Equation S1.

$$\log(Q_e - Q_t) = \log Q_e - \frac{k_1}{2.303}t \qquad (S1)$$

Where k_1 (min^{-1}) is the first-order rate constant. Pseudo-second-order rate equation based on the amount of adsorbate on the adsorbent as displayed in Equation S2.

$$\frac{t}{Q_t} = \frac{1}{k_2 Q_e^2} + \frac{1}{Q_e}t \qquad (S2)$$

Where k_2 (g/ (mg·min)) is the second-order rate constant. By plotting t/Q_t versus t, the second-order rate constant (k_2) and Q_e are obtained from intercept and slope, respectively.

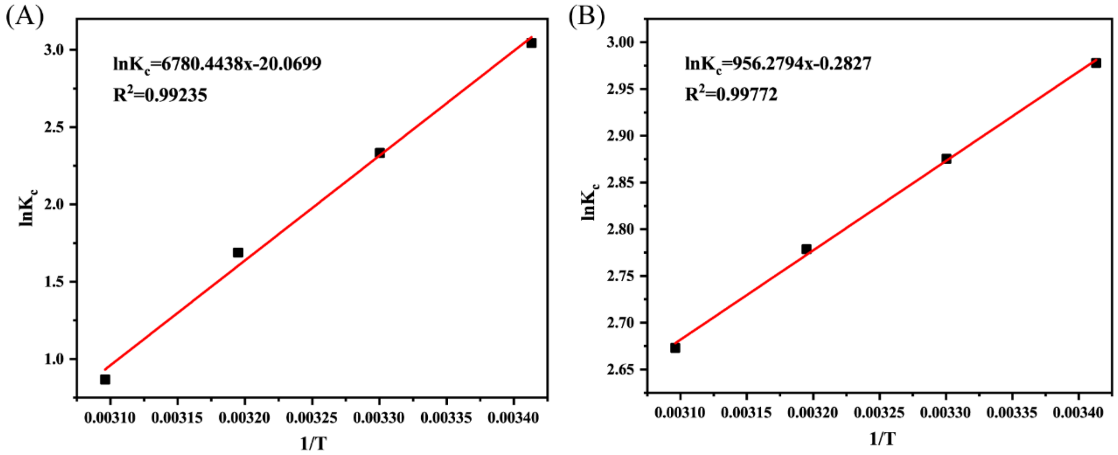

Figure S5. Adsorption thermodynamics of geopolymer: (A) M-20H/N-0.50P-3%H_2O_2 and (B) N-20H/N-0.50P-3%H_2O_2.

S2.3.3. Adsorption isotherms

L model：

$$\frac{C_e}{Q_e} = \frac{C_e}{Q_0} + \frac{1}{Q_0 K_L} \tag{S3}$$

F model：

$$\ln Q_e = \frac{1}{n} \ln C_e + \ln K_F \tag{S4}$$

T model：

$$Q_e = B \ln A + B \ln C_e \tag{S5}$$

Where Q_0 is the maximum adsorption capacity of a single molecular layer, mg/g; K_L is the Langmuir constant, L/mg; K_F is the Freundlich constant; 1/n is the adsorption index; A and B represent the isotherm constant associated to the heat of sorption (J/mole) and isotherm constant (L/g).

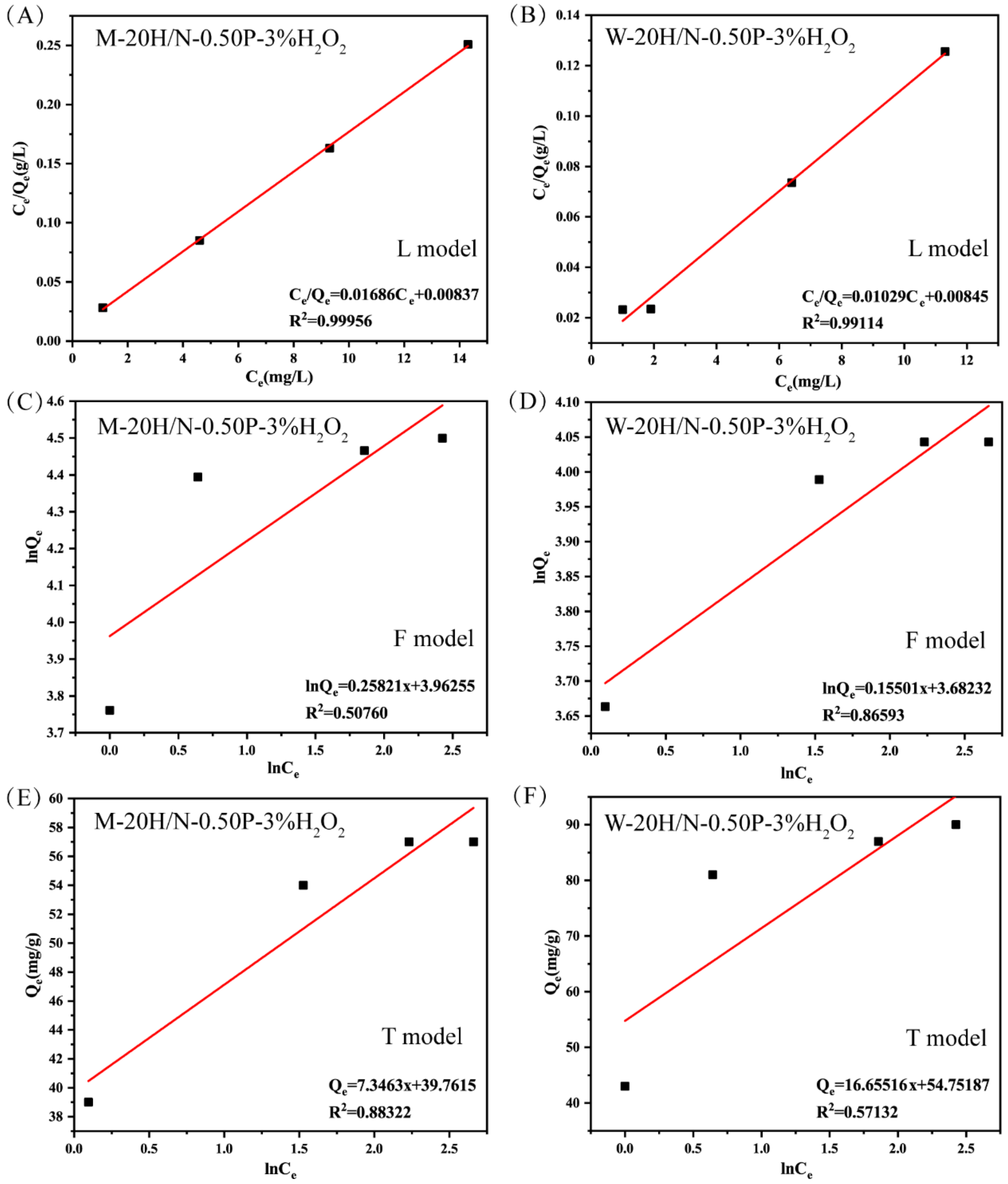

Figure S6. Corresponding adsorption isotherms for MB fitted by L model, F model, and T model of M-20H/N-0.50P-3%H_2O_2 and W-20H/N-0.50P-3%H_2O_2.

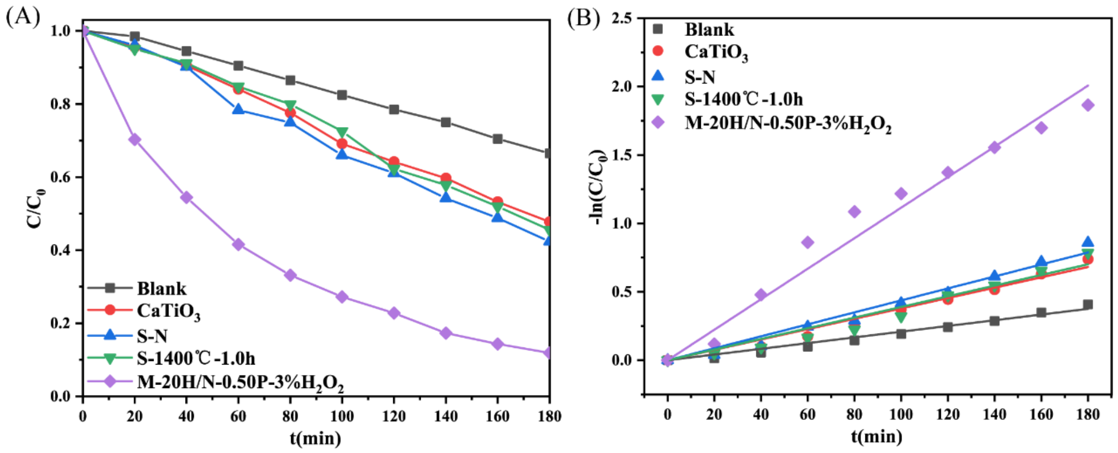

Figure S7. (A) Direct adsorption-photodegradation curves under different samples; (B) Photocatalytic degradation (-ln(C/C$_0$) against light irradiation time) of MB at 40 °C of samples with a dosage of 50 mg.

Table S2. Experimental design for adsorption tests.

	MB concentration (mg/L)	MB volume (mL)	Dosage (mg)	Temperature (℃)	Contact time (min)
Dosage	20	200	20, 30, 40, 50	40	120
Adsorption kinetics and thermodynamics	20	200	50	20, 30, 40, 50	120
Adsorption isotherms	5, 10, 15, 20	200	20	20	120
